# Gene Expression Signatures of Energetic Acclimatisation in the Reef Building Coral *Acropora millepora*

**DOI:** 10.1371/journal.pone.0061736

**Published:** 2013-05-09

**Authors:** Line K. Bay, Aurélie Guérécheau, Nikos Andreakis, Karin E. Ulstrup, Mikhail V. Matz

**Affiliations:** 1 Climate Change and Ocean Acidification Team, Australian Institute of Marine Science, Townsville, Queensland, Australia; 2 Department of Biology, University of Copenhagen, Helsingør, Denmark; 3 Integrative Biology Section, The University of Texas, Austin, Texas, United States of America; King Abdullah University of Science and Technology, Saudi Arabia

## Abstract

**Background:**

Understanding the mechanisms by which natural populations cope with environmental stress is paramount to predict their persistence in the face of escalating anthropogenic impacts. Reef-building corals are increasingly exposed to local and global stressors that alter nutritional status causing reduced fitness and mortality, however, these responses can vary considerably across species and populations.

**Methodology/Principal Findings:**

We compare the expression of 22 coral host genes in individuals from an inshore and an offshore reef location using quantitative Reverse Transcription-PCR (qRT-PCR) over the course of 26 days following translocation into a shaded, filtered seawater environment. Declines in lipid content and PSII activity of the algal endosymbionts (*Symbiodinium* ITS-1 type C2) over the course of the experiment indicated that heterotrophic uptake and photosynthesis were limited, creating nutritional deprivation conditions. Regulation of coral host genes involved in metabolism, CO_2_ transport and oxidative stress could be detected already after five days, whereas PSII activity took twice as long to respond. Opposing expression trajectories of *Tgl*, which releases fatty acids from the triacylglycerol storage, and *Dgat1*, which catalyses the formation of triglycerides, indicate that the decline in lipid content can be attributed, at least in part, by mobilisation of triacylglycerol stores. Corals from the inshore location had initially higher lipid content and showed consistently elevated expression levels of two genes involved in metabolism (aldehyde dehydrogenase) and calcification (carbonic anhydrase).

**Conclusions/Significance:**

Coral host gene expression adjusts rapidly upon change in nutritional conditions, and therefore can serve as an early signature of imminent coral stress. Consistent gene expression differences between populations indicate that corals acclimatize and/or adapt to local environments. Our results set the stage for analysis of these processes in natural coral populations, to better understand the responses of coral communities to global climate change and to develop more efficient management strategies.

## Introduction

Biotopes across the globe are increasingly affected by anthropogenic activities and changing environmental conditions [1]. On coral reefs, coral cover has declined rapidly over the past few decades (e.g., [2], [3], [4]) and up to 33% of reef-building coral species now face an elevated risk of extinction [5]. The sources of stress that underpin coral declines operate at local (e.g. eutrophication and pollution) as well as at global (e.g. elevated ocean temperature and acidification from atmospheric CO_2_) scales. The degree to which reef corals will persist into the future depends on the severity of chronic stress and the frequency of acute stress (e.g., [6], [7]–[11]). The point at which physiological tolerances are exceeded and stress occurs (e.g. bleaching) varies among coral populations and species (e.g., [9], [12], [13]). Understanding the physiological and genetic basis of this variability is a central theme in coral biology that has broad relevance to evolutionary ecology and climate change adaptation research [9], [11].

Corals are able to optimise their fitness under local environmental regimes through physiological and genetic adaptation (e.g., [14]). Physiological adaptation describes the process of tuning of the organism's physiological performance to a varying environment within its lifetime and is often interchangeably referred to as phenotypic plasticity or acclimatisation [14]–[17]. Acclimatisation can occur over relatively short time scales (typically hours to days) and, while the rate and magnitude of change is assumed to be limited by genetic make-up, it is an important physiological mechanism and can be adaptive [9], [14], [18], [19]. Stress responses and acclimatisation in corals are often inferred from changes in endosymbiotic dinoflagellate (*Symbiodinium*) densities and photosynthesis-related physiological responses. Variation in PSII performance has been documented among symbiont types [20], [21], along light and depth gradients [22]–[24], across latitudes [25], [26], historical temperature regime [27] and recent thermal history [28]–[30]. It has, however, been shown that host-specific responses to stress may occur before changes in symbiont densities or photo-physiology are evident [31]–[33]. Even though many host-specific traits are likely to play an important role in coping with chronic and acute environmental change [9], [34] our understanding of host-specific acclimatisation mechanisms remains less understood [14].

The nutritional needs of corals are met from auto and heterotrophic sources, which relative importance may vary among environmental regimes, microbial symbiont communities and species [35], [36]. The effects of auto and heterotrophy on coral physiology are complexly interlinked [37], [38]. For example, variation in light regimes and symbiont communities influence the energetic status of corals through changes in photosynthesis (e.g., [39], [40]), which can be enhanced with feeding to boost coral growth rates [38]. Energy budgets affect corals' relative allocation into skeletal and tissue growth [41], reproduction [42] and stress tolerance [43]–[45]. Environmental conditions that cause coral nutritional stress through changes in auto and heterotrophic capacity are expected to increase in the future [46], [47]. Therefore, an enhanced understanding of the physiological processes that underpin changes in coral nutritional status will therefore allow new insights into the current and future health of corals.

Gene expression is now used more commonly to understand the stress response and evolutionary ecology of corals [14], [48]. Global gene expression can elucidate how whole organisms respond to environmental stimuli and suggests molecular targets of adaptation that can be further studied with better precision using qRT-PCR [49], [50]. While post-translational changes may occur, a large proportion of differentially expressed genes (73–86%) affect downstream protein levels, and gene expression, therefore, has the potential to reveal metabolic responses to the environment [51], [52]. Analyses of changes in gene expression during metamorphosis, establishment, maintenance and stress of photosymbiosis (including bleaching) have previously been reported in corals (e.g., [53], [54], [55]). Less attention has been devoted to understanding how gene expression is affected by changes in the environment that do not cause bleaching but may affect coral physiology and condition (a.k.a. capacity adaptation [14]) but see [56]–[60]. Lastly, we are only beginning to explore how coral gene expression plasticity is modulated by genetic factors (e.g., [57], [61], [62]).

In this study, we investigate how gene expression, lipid content of the holobiont and symbiont PSII activity in corals from two source populations responded following translocation into a controlled laboratory environment with reduced potential for auto and heterotrophy for one month. Previously, we used microarray analysis to demonstrate widespread differential expression of metabolic genes and fluorescent proteins between two time points consistent with nutritional limitation [57]. Here, we extend this study to more accurately quantify expression of 25 genes implicated in metabolism, CO_2_ transport, oxidative stress, immunity and symbiosis, as well as measure photosynthesis and lipid content, over a finer timescale.

## Results

### Changes in gene expression depending on coral origin and over time

**Figure 1:**
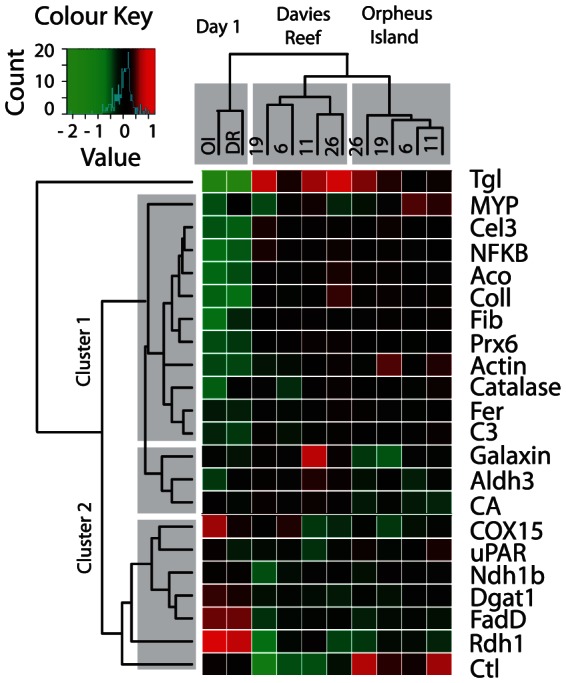
Heat map of gene expression, averaged across all samples representing each combination of factors (population and time-point). The trees correspond to hierarchical clustering of conditions (columns) and genes (rows). The conditions fall into three clusters: one uniting all initial samples taken in the field (“Day 1”, “OI” – Orpheus Island, “DR” – Davies reef), and two clusters containing subsequent common garden samples, one cluster per population. The numbers above the columns (6, 11, 19, 26) indicate days of sampling while in the common garden. The genes fall into two main clusters: the ones increasing in expression in the common garden compared to expression in the field (cluster 1), and the ones decreasing in expression (cluster 2). The color scale corresponds to log2-transformed deviance from the global mean for each gene; the maximal range of expression variation was 8-fold (*Tgl*).

The normalized relative expression of 22 target genes (Table 1) was compared between two populations from inshore (Orpheus Island, OI) and offshore (Davies Reef, DR) environments. Gene expression varied up to eight fold across the experiment and samples clustered into three groups (columns in Figure 1). The first cluster contained the field samples from both locations and the second and third clusters contained the subsequent laboratory samples separated by location. The genes (rows in Figure 1) partitioned into two main clusters based predominantly on their expression in the field. The first cluster contained 14 genes that were up-regulated after translocation into the laboratory, including two genes with roles in immunity (*C3* and *NFKB*) and three stress genes (*Fer*, *Prx6* and *Catalase*). This cluster also contained one distinctive sub-cluster of three genes associated with calcification and metabolism (i.e., Carbonic Anhydrase [*CA*], Galaxin and Aldehyde dehydrogenase [*Aldh3*]), demonstrating a tendency towards differential expression between corals from different populations (higher in Davies Reef corals, lower in Orpheus Island corals). The second cluster contained seven genes that were down-regulated in the laboratory with little differentiation between locations except C-type lectin (*Ctl)* which appeared to be up-regulated in corals from Orpheus Island in the laboratory but down-regulated in corals from Davies Reef (Figure 1). The second cluster also contained a gene with a putative role in light perception (*Rdh1*) and four metabolic genes (*Ndh1b*, *Dgat1*, *COX15* and *FadD*). Triacylglycerol lipase (*Tgl*) did not belong to any of the two clusters and displayed the greatest differential expression of all genes examined, being uniformly up-regulated in the laboratory.

**Table 1:** Genes included in the RT-qPCR assay, their abbreviations and putative function.

Protein name	Abbreviation	Putative functional role
Complement-component 3	*C3*	Essential for activating the complement system in the immune response [87], [120]
Nuclear factor Kappa-B, Subunit 2	*Nf-kb2*	Activated in response to environmental stress, pathogens and chemicals [87], [120]
Hemolytic lectin	*Cel3*	Cell surface recognition and metamorphosis [88]
C type lectin	*Ctl*	Cell surface recognition, immunity and symbiosis [88], [89]
Urokinase Plasminogen Activation Receptor	*uPar*	Serine protease involved in the plasminogen activation system [59], [95]
Superoxide dismutase	*MnSod*	Involved in the antioxidant defense system [105]
Catalase	*Cat*	Involved in the antioxidant defense system [105]
Soma ferritin	*Sof*	Involved in ferroxidase activity and cellular homeostasis by minimising Fe^2+^ availability for ROS production [100]
H+ transporting ATPase	*H-ATPase*	Involved in the ATP synthesis and photosynthesis [57], [65]
Na+/K+ exchanging ATPase	*Na,K-ATPase*	Actives transport of ions leading to a low sodium and a high potassium concentrations in cells [9]
Cytochrome c oxidase subunit XV	*Cox15*	Essential components of the mitochondrial respiratory chain (e.g., [121], [122])
Aldehyde dehydrogenase 3	*Aldh3*	Involved in the oxidation of aldehydes generated by the metabolism of a broad range of compounds, including alcohols, amino-acids, vitamins, steroids or lipids (e.g., [121], [122]
NADH dehydrogenase I chain B	*Ndh1b*	
Peroxiredoxin 6	*Prx6*	Involved in the antioxidant defense system [91]
Triacylglycerol lipase	*Tgl*	Role in releasing fatty acids from the triacylglycerol storage form [79]
Retinol dehydrogenase 1	*Rdh1*	Involved in the retinoid metabolism, vision and heat stress response [82], [83]
Diacylglycerol O-acyltransferase	*Dgat1*	Catalyzes the formation of triglycerides from diacylglycerol, using AcylCoA [79]
Acyl-CoA oxidase	*Aco*	Involved in the peroxisomal beta-oxidation of fatty acids [79]
Carbonic anhydrase	*CA*	Involved in CO2 transport associated with photosynthesis, respiration and calcification (e.g., [65])
Major yolk protein	*Myp*	Constitutes source of nutrients during gametogenesis [123], [124]
Long-chain fatty-acid-CoA ligase	*FadD*	Catalyzes the bioactivation of fatty acids, leading to the formation of acylCoA thioesters [79]
Actin1	*Actin1*	Involved in cell motility and cell structure
Collagen	*Coll*	Involved in structural support in animal tissues; dominant in mesoglea, possibly associated with symbiosis [87]
Galaxin	*Gal*	Constituent in the organic cellular matrix with a role in biomineralisation [54], [125].
Fibrinogen	*Fib*	Role in symbiosis [86]
60S Ribosomal protein L9-like isoform	*RIBOL9*	Internal control gene [105]
Unknown transcript	*Ctg1913*	Internal control gene [105]

Putative functional role is based on http://www.ncbi.nlm.nih.gov/protein, http://expasy.org/, cited sources and references therein.

**Figure 2:**
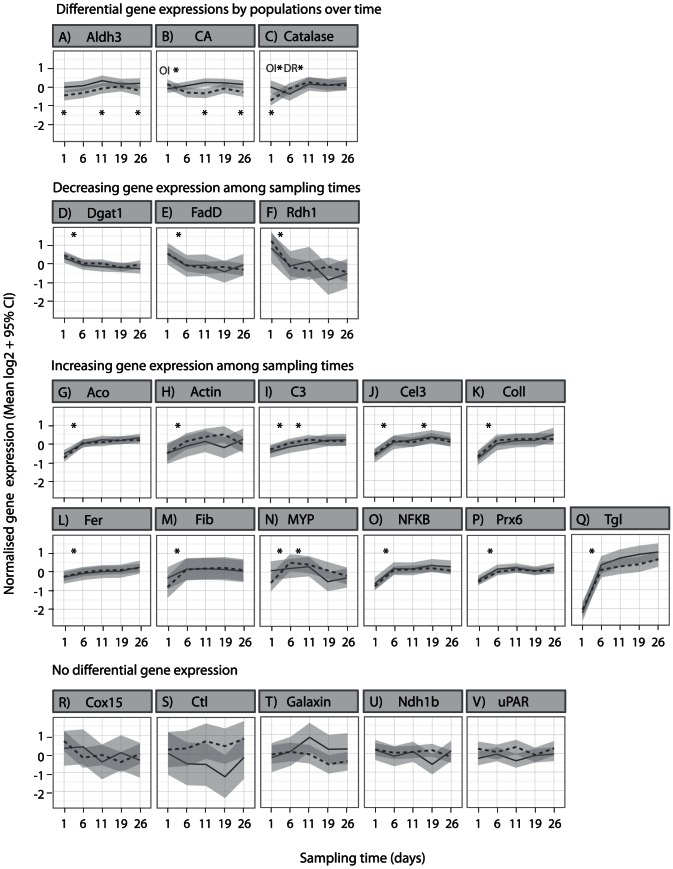
Temporal changes in gene expression of corals from two locations translocated to a common laboratory environment. Lines represent means of normalized log2 transformed expression values among replicate colonies (solid line = Davies Reef, dashed line = Orpheus Island). The grey-shaded areas represent 95% confidence intervals of means. Plots are organised by the significance of model factors (FDR adj. *P*_LRT_<0.022). Model-derived significant differences over time-points are indicated with stars above means (and are population specific in A–C, FDR adj. *P*_MCMC_<0.024 or 0.018). Significant differences between populations at specific time-points are indicated by stars below means in A–C (FDR adj. *P*_MCMC_<0.022).

Population-specific changes in gene expression over time were significant in three genes (Adj. *P*_LRT_<0.013, Fig. 2 A–C). Population-specific expression was time-dependent in *CA* and Catalase (*Cat*) (Adj. *P*_MCMC_<0.018) but not in *Aldh3*. The significance of model terms and pairwise comparisons for all genes can be found in File S1. Expression of *CA* and *Aldh3* was greater in Davies Reef corals at several sampling times whereas *Cat* was expressed at a higher level in field sampled Davies Reef corals but became similar between populations in the laboratory (Adj. *P*_MCMC_<0.022). This occurred after an initial increase in expression of Orpheus Island corals and a later decrease in Davies Reef corals (Figure 2 A–C, Adj. *P*_MCMC_<0.018).

Gene expression changed over time in 15 genes (Adj. *P*_LRT_<0.013). In three of these genes (Diacylglycerol O-acyltransferase [*Dgat1*], Long-chain fatty-acid-CoA ligase [*FadD*] and Retinol dehydrogenase [*Rdh1*]) expression declined and a significant drop in gene expression was identified between the field and the first laboratory sample (Figure 2 D–F). Gene expression increased either sharply after five days in the laboratory or gradually across the sampling times in the remaining 12 genes (Figure 2 G–Q). Variation in the expression of five genes was not significantly explained by our model terms (Figure 2 R–V).

### Changes in energetic content and photophysiology

**Figure 3:**
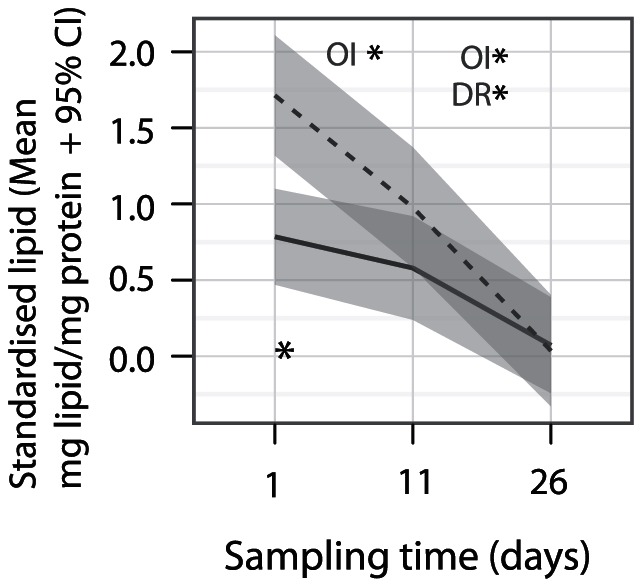
Variation in standardised lipid content of corals among sampling times and source populations. Lines represent mean standardized lipid content (mg lipid per g coral/mg protein per g coral) among replicate colonies (solid line = Davies Reef, dashed line = Orpheus Island). Grey-shaded areas represent the 95% confidence intervals of means. Population specific model-derived significant differences between consecutive sampling times are indicated above the plots (FDR adj. *P*_MCMC_<0.018) and differences between populations below the plots (FDR adj. *P*_MCMC_<0.024).

Total standardized lipid changed among sampling times and source populations (*P*_LRT_ = 0.001; Model fit in File S1). Total standardized lipid was significantly higher in Orpheus Island corals in the field and declined at approximately the same rate among sampling times. In contrast, the lipid content of Davies Reef corals was initially lower than in corals from Orpheus Island and did not decline significantly until the last sampling time (Figure 3).

**Figure 4:**
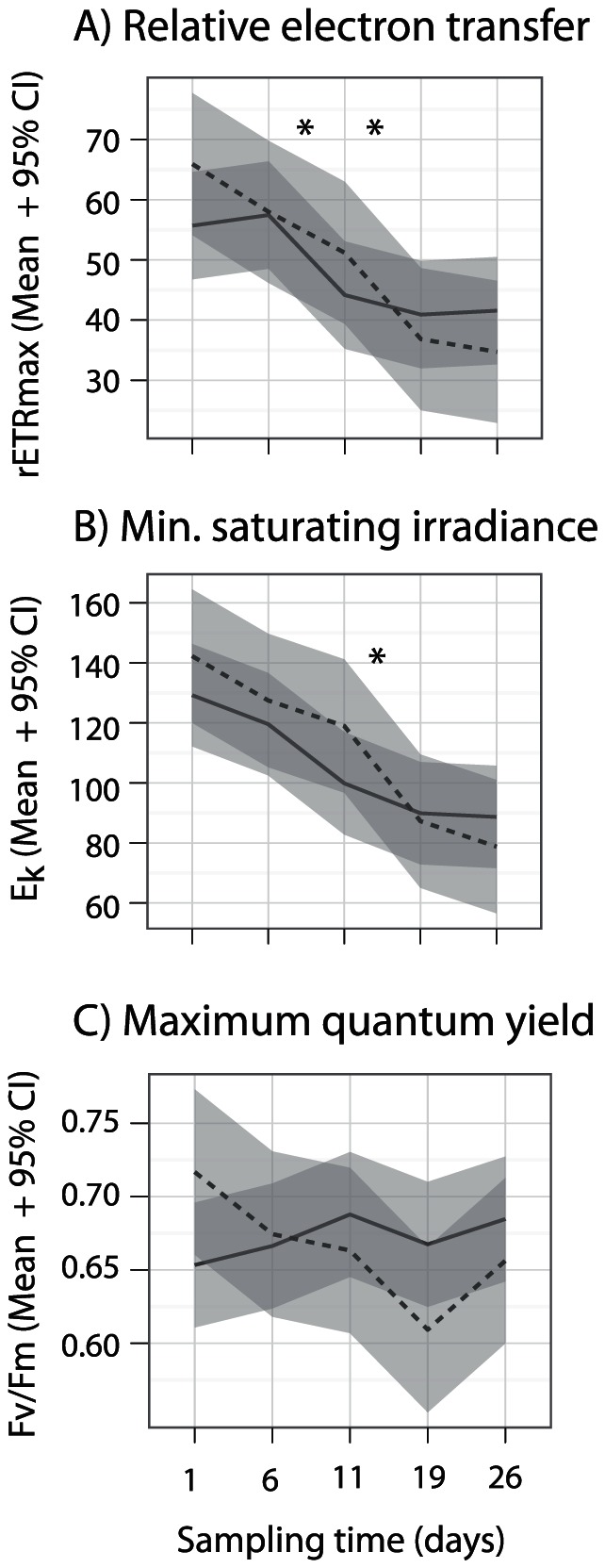
Temporal changes in photo-physiology of *Symbiodinium* C2 in corals translocated to a common laboratory environment. Panels represent A) mean relative electron transfer rate, B) mean minimum saturating irradiance and C) mean maximum quantum yield among replicate colonies (solid line = Davies Reef, dashed line = Orpheus Island). Grey-shaded areas represent the 95% confidence intervals of the means. Model-derived significant differences between consecutive sampling times are indicated above the plots (FDR adj. p<0.024).

We fitted rapid light curves to a hyperbolic tangent function to derive information about the light dependency of PSII activity of symbionts in response to the translocation into the shaded laboratory environment. Our analyses showed that relative ETR_max_ and E_k_ varied significantly over time (p<3.7×10^−8^) but not between corals sourced from different populations (Model fits in File S1). Fv/Fm consistently remained above 0.6 indicating healthy PSII function and was similar between populations, although a decline, particularly evident in Orpheus Island corals, occurred after 19 days in the laboratory (Figure 4C). The transition to lowered relative ETR_max_ and E_k_ was evident at 11 to 18 days in the shaded laboratory setting (Figure 4A–B).

## Discussion

The corals examined here displayed both immediate and longer-term responses to changing environmental conditions associated with translocation from two field locations into a shaded laboratory condition with limited potential for heterotropic feeding (i.e., supplied with 1 **µ**m filtered water). These environmentally driven changes in gene expression corresponded well with changes in physiology and many were statistically significant after five days in the laboratory. Changes in lipid content and PSII activity of symbionts were more variable and our results, therefore, add gene expression to host specific responses that can be detected earlier in the acclimatisation process compared to, for example, changes in PSII function of symbionts [31], [32].

Populations of coral may differ in their phenotype, however, the degree to which this difference is shaped by genetic or physiological adaptation is still not well understood (e.g., [14], [57], [63]). Here, three out of 22 surveyed genes showed statistically significant population-specific response to the common garden conditions. This may indicate genetic divergence, or physiological acclimatisation to the conditions at each locality. Persistent differences between populations throughout the common garden period observed for Carbonic Anhydrase (*CA*) and Aldehyde Dehydrogenase 3 (*Aldh3*) are particularly tempting to interpret as a consequence of genetic divergence between populations. In contrast, the difference in Catalase was only detectable at the initial time-point and therefore most likely reflected short-term acclimatisation to local conditions. Cytosolic α - Carbonic Anhydrase (*CA*) catalyses the reversible hydration of CO_2_ to HCO_3_^−^ (e.g., [64]) and modulates CO_2_ levels in response to respiration, photosynthesis and calcification (e.g., [65], [66]). The expression of *CA* has previously been associated with photosynthetic activity and incident light levels (e.g., [67], [68]) but did not differ between field sampled populations here, despite higher average light levels at the offshore location [69]. *CA* also did not respond negatively to lower incident light levels in the laboratory as has been shown previously [67]. *Aldh3* acts in the oxidation of aldehydes and was down-regulated in response to starvation in the liver of rats [70] and in response to low temperature acclimation in blue-fin tuna [71]. The higher expression in Davies Reef corals that also showed the slowest rate of decline in standardised total lipid may therefore suggest that the laboratory was less nutritionally challenging for this coral population compared to corals from Orpheus Island.

The energetic status of corals can be defined by the content of total or specific protein, lipid and carbohydrates and is a good indicator of coral fitness including survival following environmental stress [44], [46], [72]–[74]. Patterns of total standardised lipid content revealed here support the hypothesis that corals found the common garden conditions to be nutritionally challenging and coped by the utilisation of stored lipid reserves. We only detected three genes that consistently declined over the sampling times and two of these (*FadD*, *Dgat1*) have roles lipid metabolism [75], a process previously suggested to be under positive selection in two *Acropora* species [76]. Triacylglycerols and wax esters are the two primary classes of lipids used for storage in corals, which are specifically mobilized during bleaching [77], [78]. *Dgat1* catalyses the formation of triacylglycerols [79] and its down-regulation suggests that *A. millepora* decrease or cease storing their lipid during times of low food availability. In contrast, *Tgl*, which has the inverse role and releases fatty acids from the triacylglycerol storage form [79] increased in expression across the sampling times (Figure 2). Although declines in standardised lipid content were very variable between individual colonies and a consistent decline was only detectable in both populations after 25 days in the laboratory, the changes in expression of genes related to lipid metabolism were evident after just five days in the common garden environment (Figure 3). Because of this robust response, environmental regulation of lipid metabolism genes represent a particularly attractive avenue for further investigation of the physiological mechanisms that underpin coral energetic condition and the potential for acclimatisation in this trait.

Our results indicated no location-specific difference in photo-physiology prior to or following translocation despite large differences in the average ambient light environments. This suggests that autotrophic capacity was similar between corals from the two environments. A gradual reduction in rETRmax and Ek occurred in corals from both locations and supports acclimatisation to the shaded common laboratory environment. Shade acclimatisation is known to occur in corals exposed to low-light environments over comparable time scales [80], [81]. The expression of *CA* did not reflect patterns of PSII acclimation but was relatively constant throughout the laboratory sampling duration. In contrast, Retinol dehydrogenase (*Rdh1)* was down-regulated in corals from both locations in response to the laboratory environment. This enzyme is involved in vision in higher organisms and can respond to light environments by retinoic acid receptor activation or can act in transcriptional regulation [82], [83]. Our results suggest that *Rdhl* responds to changes in ambient light environments in a similar fashion to PSII acclimation.

Collagen (*Col*) and a Hemolytic Cel-III type lectin (*Cel3*) showed increased expression after translocation into the laboratory. *Col* is a major component of connective tissue in animals and the mesoglea in corals [84], [85]. A role of *Col* in Cnidarian symbiosis has been proposed based on expression in symbiotic vs aposymbiotic states [86] and symbiont containing cells vs those without [87]. The expression of *Ctl*, a C-type lectin was not significantly explained by our model terms, however, this gene is widely hypothesised to play a role in the onset and maintenance of symbiosis possibly through immune regulation [86], [88], [89]. It was also strongly down-regulated following acute heat stress [90] likely concomitant with changes in photosynthesis known to occur under these conditions [30]. Its expression dipped on the fourth sampling time concomitant with a decline in the maximum quantum yield (Figure 2S and 3C). Our results therefore support a role of these genes in the photo-symbiotic adaptation response of corals here (e.g., [27]), however, further research is needed to better understand the role and regulation of these genes in corals. Simultaneous investigations of host gene expression, *Symbiodinium* densities, pigment concentrations and symbiont specific gene expression will benefit future attempts to better understand how the physiological adaptation of photosymbionts affect coral gene expression, health and fitness.

Bay *et al.* [57] reported a widespread down-regulation of many metabolic genes between the field and the ten day sampling time in the laboratory, possibly indicating that metabolic arrest was occurring. Metabolic arrest is a general physiological trade-off that occurs during stress because synthesis of stress repair proteins is prioritised [91]. Here, we uncovered an up-regulation of two genes associated with immunity (*NFKB* and *C3*) and three genes associated with oxidative stress (*Fer, Prx6, Cat*), supporting the hypothesis that the laboratory treatment did impose some stress on the corals despite the absence of obvious disease or bleaching. The up-regulation of oxidate stress genes could also be a response to Reactive Oxygen Species (ROS) production as a result of increased *Tgl* activity [92]. *Mn-SOD* and *uPAR* did not respond to our experimental treatment. Urokinase Plasminogen Activation Receptor (*uPAR*) is involved in cellular signalling, adhesion, wound healing and tissue regeneration [93], [94] and expression correlates with temperature and salinity in wild corals [59]. uPAR shares a precursor with Pdcyst-rich, a gene with a putative role in calcification that was down-regulated during experimental thermal stress in *Pocillopora damicornis*, possibly because of a trade-off mechanism between growth and stress response [95].

Our results demonstrate the utility of gene expression data to detect rapid changes in physiological status as well as to understand long-term acclimatisation and/or adaptation in natural coral populations. As the co-regulation patterns and functional roles of coral genes are increasingly understood, transcriptomic data, combined with physiological and energetic analyses, will play a growing role in revealing how populations and species of corals may vary in their response to local and global stressors [49], [50], [96]. This approach will facilitate the use of coral energetic status to assess the health of coral populations and to forecast their response to stress including bleaching.

## Materials and Methods

### Sampling design

We compared the acclimatisation response of the reef building coral, *Acropora millepora*, from two source populations: Pioneer Bay, Orpheus Island (OI: 16 km off the coast and inshore, 18°36′35″S, 146°29′16″E) and Davies Reef lagoon (DR: 78 km off the coast and offshore, 18°50′11″S, 147°38′00″E) on the Great Barrier Reef, Australia. All necessary permits were obtained for the described field studies (Great Barrier Reef Marine Park Authority Permit number G06/15571.1). At offshore locations shallow corals routinely experience light levels >800 µmol photons/m^2^/s and SPM<1.5 mg/L compared with 300 µmol photons/m^2^/s and SPM<1.5 mg/L at inshore locations [69]. The laboratory environment was maintained at ambient field temperature (∼27.5°C), shaded (max. 100 µmol photons/m^2^/s) and flow-through seawater was filtered to 1 µm to remove all SPM. Three replicate branches from seven replicate colonies per population were sampled in the field and four times in the laboratory over the following month. Colonies ranged from 15 to 22 cm in diameter (mean diameter = 18.5 cm), which corresponds to ca. 225 branches. Sampled branches were removed from the central section of colonies and constituted ∼7% of colony biomass. All colonies were the same colour morph (pink) and hosted the same type of photosynthetic endosymbionts (*Symbiodinium* ITS-1 C2 *sensu* [97]). Colonies were separated by >10 meters to minimise the potential for collection of clones. The two populations we sampled were likely genetically differentiated as demonstrated with allozymes and microsatellites for nearby, and similarly spaced sampling locations [98], [99]. Corals were sampled at midday (±15 min.) for gene expression analysis and after dark for photophysiology and energetic analyses.

### Gene expression analysis

We used the GeXP analysis system (Beckman Coulter) to develop and implement a multiplex reverse transcription (RT) qPCR assay to analyse the expression of 29 genes [100]–[103]. Target genes were selected to represent a range of biological functions including metabolism, tissue and skeletal growth, immunity, symbiosis and oxidative stress (Table 1). We designed specific primers for 29 genes (26 target and 3 control genes) and optimized the assay following principles outlined in [100], [101], [104]. Amplicons were restricted to sizes of 100 to 260 base pairs (bp) and were spaced by at least five bp. We used the KanR gene as a spike-in reference gene with an amplification product of 325 bp length (File S2). Of the 29 genes included in the assay, 28 amplified consistently in all samples but VgnP did not, and was therefore eliminated from all further analyses.

The mRNA was extracted with poly-T magnetic beads using manufacturer's recommendations (Invitrogen) [105]. The concentration and purity of mRNA in each sample was evaluated using a Nanodrop spectrophotometer. Reverse transcription (RT) of mRNA to cDNA was achieved in 10 µL reaction volumes containing 10 ng sample mRNA, 2.5 µL of KanR RNA (5 ng/µL), 2 µL of RT Buffer 5X (GenomeLabTM GeXP Start Kit) and 2 µL of RT Reverse Primer Plex (Specific primer concentrations in File S2). Negative controls (no template and no reverse transcriptase) were included to test for mRNA and DNA contamination and never produced amplification products. Reverse transcription was conducted in a thermocycler (96-well Applied Biosystems 2720) at 48°C for 1 min, 37°C for 5 min, 42°C for 60 min with a final denaturation at 95°C for 5 min. To quantify technical variation associated with RT, PCR amplification and electrophoresis were performed three times on each mRNA extraction.

Polymerase Chain Reaction (PCR) was performed on cDNA in 10 µL reaction volumes containing 2 µL of PCR Buffer, 2 µL of MgCl_2_ (25 mM, ABgene), 1 µL of Forward Primer Plex (containing all forward primers at 200 nM) and 0.35 µL of Thermo-Start DNA Polymerase (ABgene). The PCR thermal cycling program included a 10 min denaturation step at 95°C, followed by 35 cycles of 30 s at 94°C, 30 s at 55°C and 1 min at 68.5°C. 1 µL of 1∶20 diluted PCR product was analysed on automated capillary sequencer (CEQ™ 8800 Genetic Analysis System, Beckman-Coulter) with 0.2 µL of ET-400 DNA size standard (Beckman-Coulter). Electrophoregrams were visualized in the GenomeLab™ Genetic Analysis System software (Beckman-Coulter), filtered then imported in GenomeLabTM eXpress Profiler software where peak binning was performed to associate fragment sizes with genes and to quantify the area under the peak used to evaluate the expression level of the associated gene [104].

We first pre-normalised the data by expressing each gene as a fraction of the internal spike-in control (i.e. Kanamycin). GeNorm was then used to determine the most stable genes for use in normalisation [106]. We identified *H+* and *Na+/K+ ATPases* and *MnSOD* as the three most stable genes, but not the *a priori* control genes (CTG_1319 and Ribol9). We then applied a normalisation factor (NF) based on the geometric mean of the expression values of these three most stable genes. The normalised gene expression ratios of remaining target genes were further analysed and the *a priori* control genes, as well as the actual normalisation genes were excluded leaving 22 genes for further analysis.

### PAM analysis

Rapid light curve analysis is a tool to assess photochemical efficiency through PSII electron flow using pulse-amplitude fluorometry (PAM) [107] and is useful for rapidly assessing photo-physiological changes [80], [108]. Three terminal branches from the centre of each coral colony were collected at dusk and a rapid light curve conducted within 1 hour of sunset in a shallow seawater bath using an Imaging-PAM fluorometer (Walz) yielding 2D images of photo-physiological parameters of interest. A single area of interest (3 mm in diameter) was selected approximately 1 cm from the tip of each branch as previous studies have shown that *Symbiodinium* may be absent or in low abundance in apical polyps [109]. Each rapid light curve comprised nine incremental 10 sec irradiance steps and photochemical efficiency of PSII was assessed at each irradiance level using the saturating pulse methodology [110]. Maximum (F_v_/F_m_) and effective quantum yields (ΔF/F_m_′) were calculated as described in Bay *et al.* [57] and used to derive the relative electron transport rate (rETR) at each irradiance step. The descriptive parameters (rETR_max_, a.u. [maximum relative electron transport rate], and E_k_, µmol photons m^−2^ s^−1^ [minimum saturating irradiance]) were modeled based on a hyperbolic tangent formula devised by Platt *et al.* [111]. The instrumental settings used were described in Bay *et al.* [57].

### Energetic content

Two branches from each colony were snap frozen in liquid nitrogen following photo-physiological analysis. Each branch was crushed into a fine powder, divided into two aliquots (20% for protein and 80% for lipid analyses), freeze-dried overnight in pre-weighed containers, then re-weighed to determine the sample dry weight. Total lipid was extracted using a Dichloromethane/Methanol extraction method, and lipid content was determined gravimetrically [112]. In brief, 2∶1 Dichloromethane/Methanol was twice added to freeze-dried samples, agitated and left overnight at 4°C then filtered using Whatman GF/C filters. To purify the extracts 0.88% KCl was added and the upper aqueous phase was removed after separation for 24 hours at 4°C. An additional two wash steps were conducted in a similar fashion with 1∶1 Methanol: Distilled Water after which extracts were dried overnight at 60°C in pre-weighed foil trays and weighed. Proteins were extracted with 0.5 M NaOH at 90°C for one hour, centrifuged for 1500 g for 10 min before supernatant was removed. Total protein content in the supernatant was quantified using a Petersen - Lowry Protein Assay (Bio-Rad DC II) using BSA as a standard. Absorbance was measured at 750 nm in a spectrophotometer (BioTek Powerwave). Three technical replicates were analysed per sample (Coefficient of Variation (CV)<10%). Lipid and protein contents were standardized to dry sample weight, after which lipid was expressed as lipid/protein and averaged across the two replicate branches per colony.

### Statistical analysis

The data were analyzed using linear mixed model approach, with population, time-point, and their interaction as fixed factors, and a specific coral colony as a scalar random factor. This approach allows a more accurate estimation of among-group variance, and is less sensitive to violations of parametric assumptions and missing data compared to repeated measures ANOVA [113], [114]. The analysis was implemented using lme4 package in R [115]. The significance of each of the fixed factors was assessed by means of likelihood ratio tests between the corresponding nested models. The Benjamini – Yekutieli correction, appropriate for dependent tests [116], [117], was used to control type 1 error rate of gene expression (FDR corrected α for 22 LogLikelihood ratio tests (LRT) = 0.0132) and photo-physiology (FDR corrected for three tests α_LRT_ = 0.027). The optimality of the selected model was also confirmed by a large weight of the Akaike information criterion. Gene expression was analyzed on a gene-by-gene basis, using log2-transformed deviances from the global mean for the particular gene as the response variable [33]. The standardized lipid, and photophysiology data were directly used as response variables.

Depending on which fixed effects were significant, we evaluated the pairwise differences between consecutive time-points from the results of Markov Chain Monte Carlo (MCMC) sampling after implementing the optimal linear mixed model in *MCMCglmm* [118]. In *MCMCglmm*, the default prior was used, corresponding to the maximum-likelihood estimates of the parameters. We compared consecutive time-points and conducted four test when only the time factor was significant (FDR corrected α_MCMC_ = 0.024) and eight tests when population specific responses were detected (FDR corrected α _MCMC_ = 0.018). We also compared populations at individual time-points when the time by population interaction was significant (i.e., five tests with FDR corrected α _MCMC_ = 0.022).

Hierarchical clustering of gene expression profiles and heatmap visualisation was done using function *heatmap.2* of the package gplots; plotting of gene expression and physiological time series was done using *ggplot* function of the ggplot2 package [33]. All the analyses were performed in R statistical software environment [119].

## Supporting Information

File S1 Likelihood ratio tests, Akaike weights and pairwise differences from gene expression and physiological analyses.
(PDF)

File S2 Genes included in the gene expression assay, abbreviations, accession numbers, concentration of primer in Reverse Primer Plex (nM), length of the transcript (bp) and Oligo sequences.
(PDF)
