# Supplementary material for: Gene Expression Signatures of Energetic Acclimatisation in the Reef Building Coral Acropora millepora
Source: PLoS One. 2013 May 9;8(5):e61736. doi: 10.1371/journal.pone.0061736 (PMC3650039; doi:10.1371/journal.pone.0061736)
Supplement: File S1 — Likelihood ratio tests, Akaike weights and pairwise differences from gene expression and physiological analyses. (PDF) [file pone.0061736.s001.pdf]

Likelihood Ratio Tests and Akaike Weights of nested models

A) Fits of five models to the expression levels of 22 genes in coral sampled from two populations in the field and four times in the laboratory. The X - axis describes the model terms (N = none, T = time, P = source population, + = additive effect, * = interaction). The y-axis describes Akaike Weights.

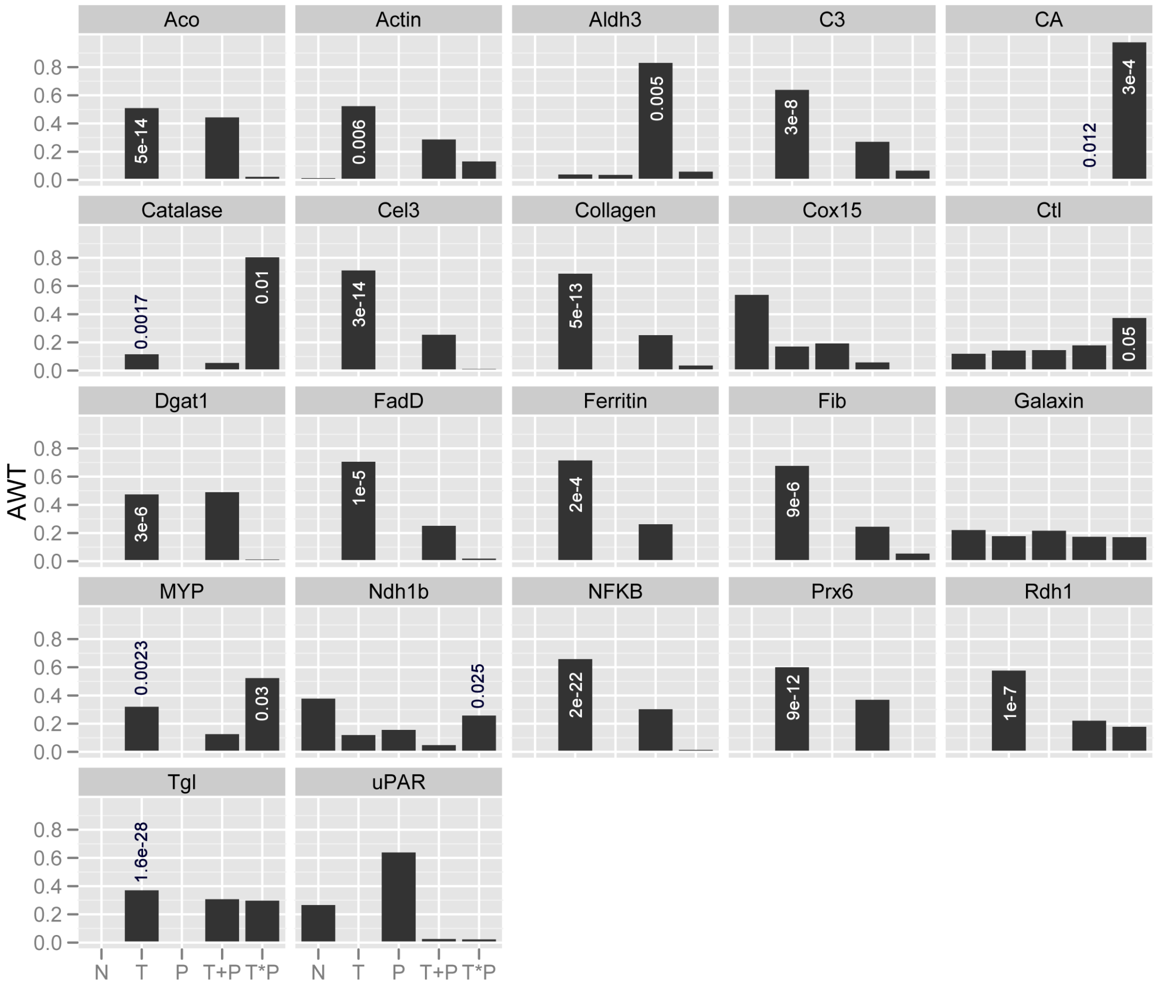

B) Fits of five models to the PSII parameters and standardised lipid content in corals sampled from two populations in the field and four times in the laboratiory. The X - axis describes the model terms (N = none, T = time, P = source population, + = additive effect, * = interaction). The y-axis describes Akaike Weights.

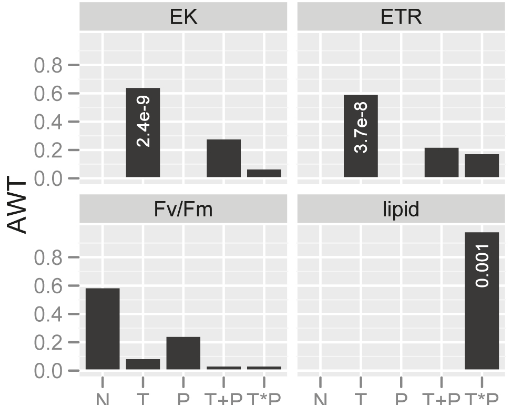

Pairwise differences in gene expression and physiology among locations and times using MCMC sampling

A) Differences in gene expression per location over time

Population	gene	t1	t2	pvalue
DR	Aco	time1	time6	<5e-04
DR	Aco	time6	time11	0.0745
DR	Aco	time11	time19	0.389
DR	Aco	time19	time26	0.154
DR	Actin	time1	time6	0.1205
DR	Actin	time6	time11	0.211
DR	Actin	time11	time19	0.154
DR	Actin	time19	time26	0.083
DR	Aldh3	time1	time6	0.3445
DR	Aldh3	time6	time11	0.0595
DR	Aldh3	time11	time19	0.1615
DR	Aldh3	time19	time26	0.5575
DR	C3	time1	time6	0.0185
DR	C3	time6	time11	0.098
DR	C3	time11	time19	0.0565
DR	C3	time19	time26	0.4455
DR	CA	time1	time6	0.115
DR	CA	time6	time11	0.1155
DR	CA	time11	time19	0.4275
DR	CA	time19	time26	0.2965
DR	Catalase	time1	time6	0.038
DR	Catalase	time6	time11	0.013
DR	Catalase	time11	time19	0.3675
DR	Catalase	time19	time26	0.2825
DR	Cel3	time1	time6	<5e-04
DR	Cel3	time6	time11	0.1575
DR	Cel3	time11	time19	0.11
DR	Cel3	time19	time26	0.1515
DR	Collagen	time1	time6	<5e-04
DR	Collagen	time6	time11	0.156
DR	Collagen	time11	time19	0.4525
DR	Collagen	time19	time26	0.032
DR	Cox15	time1	time6	0.562
DR	Cox15	time6	time11	0.0675
DR	Cox15	time11	time19	0.1785
DR	Cox15	time19	time26	0.2335
DR	CTG_1913	time1	time6	0.326
DR	CTG_1913	time6	time11	0.1175
DR	CTG_1913	time11	time19	0.035
DR	CTG_1913	time19	time26	0.0135
DR	Ctl	time1	time6	0.066
DR	Ctl	time6	time11	0.5405
DR	Ctl	time11	time19	0.0375
DR	Ctl	time19	time26	0.003
DR	Dgat1	time1	time6	0.0145
DR	Dgat1	time6	time11	0.263
DR	Dgat1	time11	time19	0.58

DR	Dgat1	time19	time26	0.327
DR	FadD	time1	time6	0.004
DR	FadD	time6	time11	0.4415
DR	FadD	time11	time19	0.0655
DR	FadD	time19	time26	0.0575
DR	Ferritin	time1	time6	0.1055
DR	Ferritin	time6	time11	0.255
DR	Ferritin	time11	time19	0.4475
DR	Ferritin	time19	time26	0.0865
DR	Fib	time1	time6	0.0165
DR	Fib	time6	time11	0.4745
DR	Fib	time11	time19	0.4195
DR	Fib	time19	time26	0.3315
DR	Galaxin	time1	time6	0.171
DR	Galaxin	time6	time11	0.0185
DR	Galaxin	time11	time19	0.0385
DR	Galaxin	time19	time26	0.427
DR	H.ATPase	time1	time6	0.094
DR	H.ATPase	time6	time11	0.0035
DR	H.ATPase	time11	time19	0.271
DR	H.ATPase	time19	time26	0.0675
DR	MYP	time1	time6	0.3245
DR	MYP	time6	time11	0.617
DR	MYP	time11	time19	0.006
DR	MYP	time19	time26	0.275
DR	Na.K.ATPase	time1	time6	0.013
DR	Na.K.ATPase	time6	time11	0.007
DR	Na.K.ATPase	time11	time19	0.3585
DR	Na.K.ATPase	time19	time26	0.1245
DR	Ndh1b	time1	time6	0.097
DR	Ndh1b	time6	time11	0.1635
DR	Ndh1b	time11	time19	0.0065
DR	Ndh1b	time19	time26	0.0075
DR	NFKB	time1	time6	<5e-04
DR	NFKB	time6	time11	0.4295
DR	NFKB	time11	time19	0.0285
DR	NFKB	time19	time26	0.231
DR	Prx6	time1	time6	<5e-04
DR	Prx6	time6	time11	0.2615
DR	Prx6	time11	time19	0.076
DR	Prx6	time19	time26	0.0565
DR	Rdh1	time1	time6	0.006
DR	Rdh1	time6	time11	0.2715
DR	Rdh1	time11	time19	0.005
DR	Rdh1	time19	time26	0.1715
DR	RIBOL9	time1	time6	0.1515
DR	RIBOL9	time6	time11	0.1625
DR	RIBOL9	time11	time19	0.149
DR	RIBOL9	time19	time26	0.298
DR	SOD.MN	time1	time6	0.087
DR	SOD.MN	time6	time11	0.1915
DR	SOD.MN	time11	time19	0.202
DR	SOD.MN	time19	time26	0.0095

DR	Tgl	time1	time6	<5e-04
DR	Tgl	time6	time11	0.0405
DR	Tgl	time11	time19	0.1725
DR	Tgl	time19	time26	0.2785
DR	uPAR	time1	time6	0.1935
DR	uPAR	time6	time11	0.076
DR	uPAR	time11	time19	0.1225
DR	uPAR	time19	time26	0.3695
OI	Aco	time1	time6	<5e-04
OI	Aco	time6	time11	0.42
OI	Aco	time11	time19	0.171
OI	Aco	time19	time26	0.531
OI	Actin	time1	time6	0.0255
OI	Actin	time6	time11	0.229
OI	Actin	time11	time19	0.339
OI	Actin	time19	time26	0.0425
OI	Aldh3	time1	time6	0.2305
OI	Aldh3	time6	time11	0.0875
OI	Aldh3	time11	time19	0.238
OI	Aldh3	time19	time26	0.082
OI	C3	time1	time6	0.004
OI	C3	time6	time11	0.0485
OI	C3	time11	time19	0.2855
OI	C3	time19	time26	0.4695
OI	CA	time1	time6	0.0025
OI	CA	time6	time11	0.361
OI	CA	time11	time19	0.0365
OI	CA	time19	time26	0.105
OI	Catalase	time1	time6	0.0025
OI	Catalase	time6	time11	0.064
OI	Catalase	time11	time19	0.259
OI	Catalase	time19	time26	0.45
OI	Cel3	time1	time6	<5e-04
OI	Cel3	time6	time11	0.7195
OI	Cel3	time11	time19	0.039
OI	Cel3	time19	time26	0.0545
OI	Collagen	time1	time6	<5e-04
OI	Collagen	time6	time11	0.3595
OI	Collagen	time11	time19	0.446
OI	Collagen	time19	time26	0.457
OI	Cox15	time1	time6	0.059
OI	Cox15	time6	time11	0.5975
OI	Cox15	time11	time19	0.237
OI	Cox15	time19	time26	0.209
OI	CTG_1913	time1	time6	0.002
OI	CTG_1913	time6	time11	0.3205
OI	CTG_1913	time11	time19	0.474
OI	CTG_1913	time19	time26	0.1215
OI	Ctl	time1	time6	0.4115
OI	Ctl	time6	time11	0.157
OI	Ctl	time11	time19	0.214
OI	Ctl	time19	time26	0.134
OI	Dgat1	time1	time6	0.0025

OI	Dgat1	time6	time11	0.5055
OI	Dgat1	time11	time19	0.1035
OI	Dgat1	time19	time26	0.169
OI	FadD	time1	time6	0.003
OI	FadD	time6	time11	0.277
OI	FadD	time11	time19	0.4005
OI	FadD	time19	time26	0.2425
OI	Ferritin	time1	time6	0.057
OI	Ferritin	time6	time11	0.2905
OI	Ferritin	time11	time19	0.4125
OI	Ferritin	time19	time26	0.231
OI	Fib	time1	time6	5.00E-04
OI	Fib	time6	time11	0.41
OI	Fib	time11	time19	0.5555
OI	Fib	time19	time26	0.309
OI	Galaxin	time1	time6	0.352
OI	Galaxin	time6	time11	0.3525
OI	Galaxin	time11	time19	0.0605
OI	Galaxin	time19	time26	0.31
OI	H.ATPase	time1	time6	0.0705
OI	H.ATPase	time6	time11	0.0175
OI	H.ATPase	time11	time19	0.3765
OI	H.ATPase	time19	time26	0.067
OI	MYP	time1	time6	5.00E-04
OI	MYP	time6	time11	0.3495
OI	MYP	time11	time19	0.171
OI	MYP	time19	time26	0.196
OI	Na.K.ATPase	time1	time6	0.0685
OI	Na.K.ATPase	time6	time11	0.016
OI	Na.K.ATPase	time11	time19	0.1025
OI	Na.K.ATPase	time19	time26	0.037
OI	Ndh1b	time1	time6	0.247
OI	Ndh1b	time6	time11	0.427
OI	Ndh1b	time11	time19	0.3675
OI	Ndh1b	time19	time26	0.0895
OI	NFKB	time1	time6	<5e-04
OI	NFKB	time6	time11	0.312
OI	NFKB	time11	time19	0.246
OI	NFKB	time19	time26	0.078
OI	Prx6	time1	time6	<5e-04
OI	Prx6	time6	time11	0.1265
OI	Prx6	time11	time19	0.235
OI	Prx6	time19	time26	0.429
OI	Rdh1	time1	time6	5.00E-04
OI	Rdh1	time6	time11	0.281
OI	Rdh1	time11	time19	0.2615
OI	Rdh1	time19	time26	0.1845
OI	RIBOL9	time1	time6	0.3305
OI	RIBOL9	time6	time11	0.0525
OI	RIBOL9	time11	time19	0.1755
OI	RIBOL9	time19	time26	0.336
OI	SOD.MN	time1	time6	0.2665
OI	SOD.MN	time6	time11	0.2205

OI	SOD.MN	time11	time19	0.128
OI	SOD.MN	time19	time26	0.1835
OI	Tgl	time1	time6	<5e-04
OI	Tgl	time6	time11	0.187
OI	Tgl	time11	time19	0.3115
OI	Tgl	time19	time26	0.099
OI	uPAR	time1	time6	0.2175
OI	uPAR	time6	time11	0.1175
OI	uPAR	time11	time19	0.0375
OI	uPAR	time19	time26	0.0685

B) Differences in gene expression over time averaged across locations

gene	t1	t2	pvalue
Aco	time1	time6	<5e-04
Aco	time11	time19	0.3335
Aco	time19	time26	0.2615
Aco	time6	time11	0.125
Actin	time1	time6	0.015
Actin	time11	time19	0.346
Actin	time19	time26	0.435
Actin	time6	time11	0.142
Aldh3	time1	time6	0.207
Aldh3	time11	time19	0.394
Aldh3	time19	time26	0.192
Aldh3	time6	time11	0.012
C3	time1	time6	0.001
C3	time11	time19	0.2545
C3	time19	time26	0.47
C3	time6	time11	0.0195
CA	time1	time6	0.119
CA	time11	time19	0.1505
CA	time19	time26	0.128
CA	time6	time11	0.281
Catalase	time1	time6	0.2235
Catalase	time11	time19	0.257
Catalase	time19	time26	0.369
Catalase	time6	time11	0.0035
Cel3	time1	time6	<5e-04
Cel3	time11	time19	0.0165
Cel3	time19	time26	0.0325
Cel3	time6	time11	0.3765
Collagen	time1	time6	<5e-04
Collagen	time11	time19	0.4635
Collagen	time19	time26	0.12
Collagen	time6	time11	0.164
Cox15	time1	time6	0.1315
Cox15	time11	time19	0.444
Cox15	time19	time26	0.4905
Cox15	time6	time11	0.2
CTG_1913	time1	time6	0.017
CTG_1913	time11	time19	0.1145
CTG_1913	time19	time26	0.2375

CTG_1913	time6	time11	0.13
Ctl	time1	time6	0.196
Ctl	time11	time19	0.039
Ctl	time19	time26	0.0055
Ctl	time6	time11	0.259
Dgat1	time1	time6	0.002
Dgat1	time11	time19	0.138
Dgat1	time19	time26	0.342
Dgat1	time6	time11	0.3095
FadD	time1	time6	<5e-04
FadD	time11	time19	0.1805
FadD	time19	time26	0.261
FadD	time6	time11	0.374
Ferritin	time1	time6	0.022
Ferritin	time11	time19	0.4005
Ferritin	time19	time26	0.0735
Ferritin	time6	time11	0.184
Fib	time1	time6	<5e-04
Fib	time11	time19	0.5045
Fib	time19	time26	0.256
Fib	time6	time11	0.4175
Galaxin	time1	time6	0.173
Galaxin	time11	time19	0.014
Galaxin	time19	time26	0.4285
Galaxin	time6	time11	0.1135
H.ATPase	time1	time6	0.026
H.ATPase	time11	time19	0.3875
H.ATPase	time19	time26	0.5035
H.ATPase	time6	time11	<5e-04
MYP	time1	time6	0.006
MYP	time11	time19	0.0115
MYP	time19	time26	0.4295
MYP	time6	time11	0.495
Na.K.ATPase	time1	time6	0.004
Na.K.ATPase	time11	time19	0.117
Na.K.ATPase	time19	time26	0.0125
Na.K.ATPase	time6	time11	0.001
Ndh1b	time1	time6	0.093
Ndh1b	time11	time19	0.065
Ndh1b	time19	time26	0.1735
Ndh1b	time6	time11	0.23
NFKB	time1	time6	<5e-04
NFKB	time11	time19	0.0275
NFKB	time19	time26	0.057
NFKB	time6	time11	0.369
Prx6	time1	time6	<5e-04
Prx6	time11	time19	0.0605
Prx6	time19	time26	0.108
Prx6	time6	time11	0.1055
Rdh1	time1	time6	<5e-04
Rdh1	time11	time19	0.0675
Rdh1	time19	time26	0.5515
Rdh1	time6	time11	0.526

RIBOL9	time1	time6	0.1555
RIBOL9	time11	time19	0.086
RIBOL9	time19	time26	0.478
RIBOL9	time6	time11	0.02
SOD.MN	time1	time6	0.0835
SOD.MN	time11	time19	0.083
SOD.MN	time19	time26	0.0095
SOD.MN	time6	time11	0.119
Tgl	time1	time6	<5e-04
Tgl	time11	time19	0.162
Tgl	time19	time26	0.097
Tgl	time6	time11	0.033
uPAR	time1	time6	0.4195
uPAR	time11	time19	0.3385
uPAR	time19	time26	0.11
uPAR	time6	time11	0.385

C) Difference in gene expression between locations at specific times

gene	time	pvalue
Aco	time1	0.084
Aco	time11	0.187
Aco	time19	0.413
Aco	time26	0.1765
Aco	time6	0.373
Actin	time1	0.475
Actin	time11	0.257
Actin	time19	0.021
Actin	time26	0.215
Actin	time6	0.2235
Aldh3	time1	0.0105
Aldh3	time11	0.014
Aldh3	time19	0.247
Aldh3	time26	0.02
Aldh3	time6	0.0275
C3	time1	0.2695
C3	time11	0.1345
C3	time19	0.575
C3	time26	0.3495
C3	time6	0.1685
CA	time1	0.069
CA	time11	5.00E-04
CA	time19	0.0335
CA	time26	0.01
CA	time6	0.023
Catalase	time1	5.00E-04
Catalase	time11	0.323
Catalase	time19	0.4255
Catalase	time26	0.282
Catalase	time6	0.083
Cel3	time1	0.3695
Cel3	time11	0.304
Cel3	time19	0.408

Cel3	time26	0.303
Cel3	time6	0.342
Collagen	time1	0.3195
Collagen	time11	0.3595
Collagen	time19	0.355
Collagen	time26	0.1505
Collagen	time6	0.202
Cox15	time1	0.223
Cox15	time11	0.2615
Cox15	time19	0.162
Cox15	time26	0.2615
Cox15	time6	0.139
CTG_1913	time1	0.193
CTG_1913	time11	0.2685
CTG_1913	time19	0.003
CTG_1913	time26	0.198
CTG_1913	time6	0.0675
Ctl	time1	0.386
Ctl	time11	0.0555
Ctl	time19	0.0145
Ctl	time26	0.0925
Ctl	time6	0.1275
Dgat1	time1	0.215
Dgat1	time11	0.155
Dgat1	time19	0.4795
Dgat1	time26	0.089
Dgat1	time6	0.308
FadD	time1	0.4825
FadD	time11	0.3115
FadD	time19	0.228
FadD	time26	0.205
FadD	time6	0.4665
Ferritin	time1	0.5275
Ferritin	time11	0.384
Ferritin	time19	0.4
Ferritin	time26	0.42
Ferritin	time6	0.372
Fib	time1	0.116
Fib	time11	0.499
Fib	time19	0.429
Fib	time26	0.4405
Fib	time6	0.5145
Galaxin	time1	0.3455
Galaxin	time11	0.022
Galaxin	time19	0.039
Galaxin	time26	0.108
Galaxin	time6	0.4805
H.ATPase	time1	0.1385
H.ATPase	time11	0.094
H.ATPase	time19	0.226
H.ATPase	time26	0.0045
H.ATPase	time6	0.1545
MYP	time1	0.0245

MYP	time11	0.366
MYP	time19	0.04
MYP	time26	0.3775
MYP	time6	0.1915
Na.K.ATPase	time1	0.2885
Na.K.ATPase	time11	0.0925
Na.K.ATPase	time19	0.26
Na.K.ATPase	time26	0.117
Na.K.ATPase	time6	0.143
Ndh1b	time1	0.5215
Ndh1b	time11	0.4825
Ndh1b	time19	0.0135
Ndh1b	time26	0.171
Ndh1b	time6	0.269
NFKB	time1	0.277
NFKB	time11	0.469
NFKB	time19	0.2415
NFKB	time26	0.16
NFKB	time6	0.368
Prx6	time1	0.2675
Prx6	time11	0.2835
Prx6	time19	0.518
Prx6	time26	0.1585
Prx6	time6	0.157
Rdh1	time1	0.2175
Rdh1	time11	0.151
Rdh1	time19	0.0575
Rdh1	time26	0.5365
Rdh1	time6	0.4485
RIBOL9	time1	0.481
RIBOL9	time11	0.526
RIBOL9	time19	0.4345
RIBOL9	time26	0.349
RIBOL9	time6	0.3965
SOD.MN	time1	0.3675
SOD.MN	time11	0.408
SOD.MN	time19	0.489
SOD.MN	time26	0.1855
SOD.MN	time6	0.563
Tgl	time1	0.2855
Tgl	time11	0.073
Tgl	time19	0.0405
Tgl	time26	0.105
Tgl	time6	0.1745
uPAR	time1	0.03
uPAR	time11	0.0065
uPAR	time19	0.542
uPAR	time26	0.128
uPAR	time6	0.3245

D) Differences in PAM measurements over time (averaged across locations)

PAM measure	t1	t2	pvalue
ek	time1	time6	0.0755
ek	time1	time11	5.00E-04
ek	time1	time19	<5e-04
ek	time1	time26	<5e-04
ek	time6	time11	0.04
ek	time6	time19	<5e-04
ek	time6	time26	<5e-04
ek	time11	time19	0.0055
ek	time11	time26	<5e-04
ek	time19	time26	0.295
etr	time1	time6	0.221
etr	time1	time11	0.002
etr	time1	time19	<5e-04
etr	time1	time26	<5e-04
etr	time6	time11	0.0065
etr	time6	time19	<5e-04
etr	time6	time26	<5e-04
etr	time11	time19	0.0155
etr	time11	time26	0.0075
etr	time19	time26	0.5735
fvfm	time1	time6	0.2695
fvfm	time1	time11	0.3475
fvfm	time1	time19	0.0325
fvfm	time1	time26	0.2845
fvfm	time6	time11	0.5885
fvfm	time6	time19	0.0925
fvfm	time6	time26	0.4995
fvfm	time11	time19	0.0675
fvfm	time11	time26	0.426
fvfm	time19	time26	0.1025

E) Differences in lipid content of locations over time

pop	t1	t2	pvalue
dr	time1	time10	0.1975
dr	time1	time26	0.003
dr	time10	time26	0.0175
oi	time1	time10	0.0015
oi	time1	time26	<5e-04
oi	time10	time26	<5e-04

F) Differences in lipid content between locations

time	pvalue
time1	5.00E-04
time10	0.0595
time26	0.43
