# Supplementary material for: Gene Expression Signatures of Energetic Acclimatisation in the Reef Building Coral Acropora millepora
Source: PLoS One. 2013 May 9;8(5):e61736. doi: 10.1371/journal.pone.0061736 (PMC3650039; doi:10.1371/journal.pone.0061736)
Supplement: File S2 — Genes included in the gene expression assay, abbreviations, accession numbers, concentration of primer in Reverse Primer Plex (nM), length of the transcript (bp) and Oligo sequences. (PDF) [file pone.0061736.s002.pdf]

Supporting Information File S2: Genes included in the gene expression assay, abbreviations, accession numbers, concentration of primer in Reverse Primer Plex (nM), length of the transcript (bp) and Oligo sequences.

Protein name	Abbreviation	Acc. num.	Conc	Bp	Oligo sequences
Complement-component 3	*C3*	EF090257	50	137	L: AGGTGACACTATAGAATAAGTTTCTCGTGGTCGCATTT<br>R: GTACGACTCACTATAGGGATGATGGAGACATTTGAGGGG
Nuclear factor Kappa-B, Subunit 2	*Nf-kb2*	EZ002200	500	197	L: AGGTGACACTATAGAATAAAATCCAATGCCTCATGCTC<br>R: GTACGACTCACTATAGGGATCTGTCCATCAACACCAAGC
Hemolytic lectin	*Cel3*	GO003659	500	187	L: AGGTGACACTATAGAATATGGCAGGAAGCAAGAGAAAT<br>R: GTACGACTCACTATAGGGATGCCAGTAGTTTCCCTCTGG
C type lectin	*Ctl*	GO001638	500	237	L: AGGTGACACTATAGAATAAACAGGTGTTCGTCTCGTCC<br>R: GTACGACTCACTATAGGGAACGACTTTGTGCTGGCTCTT
Urokinase	*Upar*	SeqIndex3826	1000	147	L: AGGTGACACTATAGAATATGAGAGCTCGAAGAGTTGGA<br>R: GTACGACTCACTATAGGGATGCTGTCCCTGTTTTCTGTG
Superoxide dismutase	*MnSod*	DY581262	500	172	L: AGGTGACACTATAGAATAATGTTGTGCCGTAGTGTTCG<br>R: GTACGACTCACTATAGGGAGATTGCAGGTTTCAAGGCAT
Catalase	*Cat*	EZ013640	500	177	L: AGGTGACACTATAGAATAATCCGCTGATTACTGTTGGC<br>R: GTACGACTCACTATAGGGAGCGACCCTGCAACATCTTAT
Soma ferritin	*Sof*	DY580747	500	182	L: AGGTGACACTATAGAATAGTGGCTGACACTCATGGAGA<br>R: GTACGACTCACTATAGGGACAAACTGGTATTCGCCCAGT
H+ transporting ATPase	*H-ATPase*	GO004405	500	167	L: AGGTGACACTATAGAATACTTTTTGGTGGAGCTGGTGT<br>R: GTACGACTCACTATAGGGAACAAATCGTTTCCTTCACGG
Na+/K+ exchanging ATPase	*Na,K-ATPase*	DY580462	500	157	L: AGGTGACACTATAGAATATGGCTATGACGAAGGGAAAC<br>R: GTACGACTCACTATAGGGATCGCTTTGATCCTTTTCGTT
Cytochrome c oxidase subunit XV	*Cox15*	DY578450	1000	142	L: AGGTGACACTATAGAATACAAACCCCTTGCTTGGATAA<br>R: GTACGACTCACTATAGGGAGGAGCAACTGAAGCATCCAT
Aldehyde dehydrogenase 3	*Aldh3*	DY579229	500	232	L: AGGTGACACTATAGAATAATGTGGAGTTCAGGCGATGT<br>R: GTACGACTCACTATAGGGAGGCATTCAGGTGTAACCCAT
NADH dehydrogenase I chain B	*Ndh1b*	DY584577	1000	297	L: AGGTGACACTATAGAATAAGAACCGGTTGCAGTGTTTT<br>R: GTACGACTCACTATAGGGATGCATCATTTCAACAGCACA
Peroxiredoxin 6	*Prx6*	DY580542	1000	192	L: AGGTGACACTATAGAATAAATGGTGAACCTTGGAGACG<br>R: GTACGACTCACTATAGGGAACACACGTCCCAACTCTGTG

Triacylglycerol lipase	*Tgl*	GO004043	1000	222	L: AGGTGACACTATAGAATAGTTGGAACATTGCGGAGAAT<br>R: GTACGACTCACTATAGGGAGACCAAAGAGGCACAAAGGA
Retinol dehydrogenase 1	*Rdh1*	GO002865	500	272	L: AGGTGACACTATAGAATAAAATTCCCTCCCATCCTTGT<br>R: GTACGACTCACTATAGGGAGCTCGCCTCTGATTTCAAAC
Diacylglycerol O-acyltransferase	*Dgat1*	DY578905	500	152	L: AGGTGACACTATAGAATATCAACCAAGTCAGCAATCCA<br>R: GTACGACTCACTATAGGGAAGCTTGGGTGAAATCCCTCT
Acyl-CoA oxidase	*Aco*	DY580294	1000	202	L: AGGTGACACTATAGAATATATTTGCTGCACACGAGCTT<br>R: GTACGACTCACTATAGGGACAAAGCAGCCAACATCCTCT
Carbonic anhydrase	*CA*	GO000656	500	212	L: AGGTGACACTATAGAATAACCTACGATGGATCGCTGAC<br>R: GTACGACTCACTATAGGGACTTCGAGAATGCACTGGACA
Major yolk protein	*Myp*	SeqIndex3069	500	227	L: AGGTGACACTATAGAATAAAAGGATGGCCACAGAGTTG<br>R: GTACGACTCACTATAGGGAAATTTCTCAGCCATGATCGG
Vitellogenin precursor	*VgnP*	GO000214	1000	242	L: AGGTGACACTATAGAATAACCTTTGTTGGCTGGATTTG<br>R: GTACGACTCACTATAGGGAGTGTGGCATTTGTGGAGACA
Long-chain fatty-acid-CoA ligase	*FadD*	DY586156	500	277	L: AGGTGACACTATAGAATATTACGGAAGCTGTGCTGAAA<br>R: GTACGACTCACTATAGGGATGGACATAAAAACCCCGAAG
Actin1	*Actin1*	SeqIndex13222	200	247	L: AGGTGACACTATAGAATACCCAGAGGCTATGTTTCAGC<br>R: GTACGACTCACTATAGGGAATCTTCATGGTTGGTGGAGC
Collagen	*Coll*	SeqIndex9903	500	252	L: AGGTGACACTATAGAATAAATAAAGGACCAGGAGCCGT<br>R: GTACGACTCACTATAGGGATCACGACAAGTCTTTGCTGG
Galaxin	*Gal*	SeqIndex676	1000	162	L: AGGTGACACTATAGAATAAGGAGAGACGCCCATAGTGA<br>R: GTACGACTCACTATAGGGATTTCCGTGACAGCACATCTC
Fibrinogen	*Fib*	SeqIndex2262	1000	267	L: AGGTGACACTATAGAATATCCAAAACAACACCTGCAAA<br>R: GTACGACTCACTATAGGGAGCATTGCTTTCCCGAGTATC
60S Ribosomal protein L9-like isoform	*RIBOL9*	DY586572	1000	257	L: AGGTGACACTATAGAATAAGAATGTCACCGTCAAGGCT<br>R: GTACGACTCACTATAGGGATTGTAGCCATAGACGACCCC
Unknown transcript	*Ctg1913*	DY585358	500	262	L: AGGTGACACTATAGAATATACCGCTACCGTAACCAAGG<br>R: GTACGACTCACTATAGGGATCAGTGCTTCACTGGCATTC
